# [FeFe]-Hydrogenase Abundance and Diversity along a Vertical Redox Gradient in Great Salt Lake, USA

**DOI:** 10.3390/ijms151221947

**Published:** 2014-11-28

**Authors:** Eric S. Boyd, Trinity L. Hamilton, Kevin D. Swanson, Alta E. Howells, Bonnie K. Baxter, Jonathan E. Meuser, Matthew C. Posewitz, John W. Peters

**Affiliations:** 1 Department of Microbiology and Immunology, Montana State University, Bozeman, MT 59717, USA; E-Mail: eboyd@montana.edu; 2 Department of Chemistry and Biochemistry, Montana State University, Bozeman, MT 59717, USA; E-Mails: tlh42@psu.edu (T.L.H.); kdswanson@live.com (K.D.S.), aehowell@asu.edu (A.E.H.); 3 Department of Biology and the Great Salt Lake Institute, Westminster College, Salt Lake City, UT 84105, USA; E-Mail: bbaxter@westminstercollege.edu; 4 Department of Chemistry and Geochemistry, Colorado School of Mines, Golden, CO 80401, USA; E-Mails: jmeuser@syntheticgenomics.com (J.E.M.), mposewit@mines.edu (M.C.P.)

**Keywords:** [FeFe]-hydrogenase, electron bifurcation, hydrogen, photosynthesis, fermentation, oxygen tolerance, hydropathy, hypersaline

## Abstract

The use of [FeFe]-hydrogenase enzymes for the biotechnological production of H_2_ or other reduced products has been limited by their sensitivity to oxygen (O_2_). Here, we apply a PCR-directed approach to determine the distribution, abundance, and diversity of *hydA* gene fragments along co-varying salinity and O_2_ gradients in a vertical water column of Great Salt Lake (GSL), UT. The distribution of *hydA* was constrained to water column transects that had high salt and relatively low O_2_ concentrations. Recovered HydA deduced amino acid sequences were enriched in hydrophilic amino acids relative to HydA from less saline environments. In addition, they harbored interesting variations in the amino acid environment of the complex H-cluster metalloenzyme active site and putative gas transfer channels that may be important for both H_2_ transfer and O_2_ susceptibility. A phylogenetic framework was created to infer the accessory cluster composition and quaternary structure of recovered HydA protein sequences based on phylogenetic relationships and the gene contexts of known complete HydA sequences. Numerous recovered HydA are predicted to harbor multiple *N*- and *C*-terminal accessory iron-sulfur cluster binding domains and are likely to exist as multisubunit complexes. This study indicates an important role for [FeFe]-hydrogenases in the functioning of the GSL ecosystem and provides new target genes and variants for use in identifying O_2_ tolerant enzymes for biotechnological applications.

## 1. Introduction

Hydrogen (H_2_) is a diffusible electron carrier with the highest energy content per unit mass of all naturally occurring fuels, and forms the basis of numerous interspecies interactions in natural microbial communities [1,2]. [NiFe]- and [FeFe]-hydrogenase, which differ in the metal composition of their respective active-site clusters, are principally responsible for H_2_ cycling in natural environments. In general, [FeFe]-hydrogenases are found in anaerobic bacteria and are especially prevalent among fermentative organisms (e.g., firmicutes) where they typically function in the regeneration of reduced electron carriers (NAD(P)H, ferredoxin (Fd)) coupled to the reduction of protons. They are also found in a number of lower eukaryotes including algae and protists, but surprisingly they have yet to be found in cyanobacteria or in archaea [3]. In contrast, [NiFe]-hydrogenases, which are primarily associated with H_2_ oxidation in energy yielding processes, are frequently found in archaea and bacteria [4,5].

The majority of H_2_ in natural environments is produced through fermentative processes [6] or through biological N_2_ fixation [7]. While both [NiFe]- and [FeFe]-hydrogenases can function in the generation of H_2_ during fermentation [3,4,5], [FeFe]-hydrogenases are generally more efficient H_2_-producing catalysts [8] and have been the subject of extensive biochemical and structural characterization in an effort to optimize these enzymes for efficient bio-H_2_ production [9,10,11,12,13]. The best characterized [FeFe]-hydrogenases are monomeric, Fd-dependent enzymes [3,5]. Examples of these enzymes include those from eukaryotic algae such as *Chlamydomonas reinhardtii* [14,15,16] and clostridial species such as *Clostridium pasteurianum* [10,17,18]. [FeFe]-hydrogenase diversity is manifested mainly in the number and type of accessory cluster domains that complement the active site H-cluster domain, the latter of which is universally conserved in currently sequenced homologs [3,19]. [FeFe]-hydrogenases from *C. reinhardtii* are the simplest and exist without additional accessory clusters. In contrast, [FeFe]-hydrogenase from *C. pasteurianum* have three additional iron-sulfur (Fe-S) cluster binding domains in the *N*-terminus which presumably function to allow coupling with external electron donors and/or acceptors. Characterization of [FeFe]-hydrogenase homologs in available genome sequences [3,19] noted substantial variation in *N*- and *C*-terminal Fe-S cluster and accessory cofactor binding motifs, suggesting potential interactions with a variety of other redox partners. In addition, such analyses performed in conjunction with an evaluation of gene context and biochemical characterization revealed differences in [FeFe]-hydrogenase quaternary structure, including the identification of multimeric homologs that likely form dimers, trimers, or tetramers [3,19].

Biochemical characterization of multimeric [FeFe]-hydrogenase enzymes from *Desulfovibrio* *fructosovorans* [20], *Thermoanaerobacter* *tengcongensis* [21], and *Thermotoga* *maritima* [22] indicate that they are pyridine nucleotide (NAD(P)H- or NADH-) linked H_2_ producing enzymes. The trimeric hydrogenase from *T. maritima* was shown to require both reduced Fd and NADH for H_2_ production [23]. Conversely, the [FeFe]-hydrogenase from *Acetobacterium* *woodii* simultaneously reduces NAD^+^ and Fd during H_2_ oxidation. The key to the reversible oxidation of H_2_ and simultaneous NAD^+^ and Fd reduction is the coupling of their energetics; the exergonic reduction of NAD^+^ by electrons derived from H_2_ allows the endergonic reduction of Fd in a process now termed electron bifurcation [24,25]. Bifurcating [FeFe]-hydrogenanse enzymes function to create electrons with a very low redox potential by simultaneously creating an electron with a high redox potential [23,25,26,27]. While the aforementioned examples indicate a directionality in the bifurcating process, [FeFe]-hydrogenases that bifurcate can in principle function *in vivo* in both the production and oxidation of H_2_, as was recently shown for a bifurcating hydrogenase in *Moorella* *thermoacetica* [27]. During growth on glucose, *M. thermoacetica* can intermittently produce H_2_ from Fd and NADH via the bifurcating hydrogenase. Likewise, this same hydrogenase can utilize produced H_2_ and CO_2_ to preform acetogenesis. Although *M. thermoacetica* does not grow well autotrophically on H_2_ and CO_2_, it uses the bifurcating hydrogenase to produce reduced Fd to drive acetogenesis. The reversible production of such low potential metabolic electrons through the process of H_2_ dependent electron bifurcation is of significant biotechnological interest since they could be modulated *in vivo* and directed toward the controlled production of highly reduced biofuel products.

Multimeric bifurcating [FeFe]-hydrogenase complexes now appear to be commonplace among anaerobic bacteria [3,19]. However, like the monomeric forms of [FeFe]-hydrogenase [28,29,30], multimeric enzymes are O_2_ sensitive which presents challenges in their application in large-scale production efforts [31]. Numerous biochemical and structural studies have been conducted to shed light on the basis of the O_2_ sensitivity of [FeFe]-hydrogenase [28,29,32,33,34,35,36,37]. One approach to address this problem is gene shuffling or randomized mutation, which generates a diverse recombinant hydrogenase library to screen for enhanced O_2_ tolerance and/or stability [12,13,38,39]. Another method is to examine the natural diversity of these enzymes across geochemical gradients (e.g., O_2_) through the use of PCR or metagenomic-directed approaches [40,41,42,43,44]. A previous application of a PCR-directed approach targeting a fragment of the gene encoding the large subunit (*hydA*) of [FeFe]-hydrogenase in a phototrophic mat community identified significant variation in the amino acid environment of the active site H-cluster [41] and previously implicated gas channels [39,45,46,47,48]. Substitutions in the amino acids lining these gas channels have been previously shown to support increased O_2_ tolerance [39,48,49]. This may suggest that natural sequence variations that would adjust the size of this gas channel may provide a biomarker for O_2_ tolerant [FeFe]-hydrogenase. While the functional implications of these substitutions/insertions have yet to be fully characterized biochemically, such findings provide impetus to continue searching natural systems for potential variants capable of tolerating higher O_2_ concentrations.

Here, we apply a PCR-directed approach to determine the distribution, abundance, and diversity of *hydA* gene fragments in a vertical water column environment of Great Salt Lake (GSL), UT that exhibits strong and co-varying gradients in salinity, O_2_, photosynthetically active radiation (PAR), pH, and temperature [50]. A phylogenetic framework was created for use in predicting the accessory cluster composition and quaternary structure of inferred HydA protein sequences obtained along the vertical gradient. The results indicate an abundance of multimeric [FeFe]-hydrogenase that are putatively involved in electron bifurcating processes. Recovered HydA protein fragments revealed evidence of adaptation to elevated salt concentrations and harbored variations in regions of the protein that form gas channels leading to the O_2_-labile FeS cubane of the active site. Similarly, novel substitutions were identified in residues that coordinate the O_2_-labile active site. This study demonstrates that enzyme variants with desired properties can potentially be recovered by examining protein diversity in microbial assemblages that have evolved in the presence of a particular environmental stress.

## 2. Results

### 2.1. Water Column Chemistry

Strong and often co-varying physical and chemical gradients were observed in the Utah Division of Wildlife Resources sample site number 3 (DWR3) vertical water column (Table 1). Salinity did not vary substantially over the interval from the surface to a depth of 6.0 meters but increased from 126 parts per thousand (ppt) at 6.0 meters to 203 and 247 ppt at depths of 6.5 and 8.0 meters, respectively. Likewise, water column temperature was nearly constant (23–24 °C) over the 0.0 to 6.0 meters vertical transect, but decreased abruptly at a depth of 6.5 and 8.0 meters (20 and 17 °C, respectively), indicating the presence of an inverted thermocline between depth intervals of 6.0 and 8.0 meters. pH varied from 5.95 to 8.05 over the eight meter depth, with the highest pH observed at a depth of 6.0 meters and the lowest pH observed at 8.0 meters. PAR decreased systematically from 1800 µmol·photons·m^−2^·s^–1^ just below the surface to 175 µmol·photons·m^−2^·s^–1^ at a depth of 4.0 meters and was below detection at a depth of 8.0 meters. Dissolved O_2_ varied little over the 0.0 to 4.0 meter vertical transect, but decreased substantially from 5.1 to 2.3 mg·L^–1^ over the 4.0 to 6.0 meters depth transect.

**Table 1:** Geographical, physical, and chemical data for the water column depths sampled ^a^.

Depth (m)	Latitude	Longitude	Salinity (ppt)	Dissolved	PAR (µmol·photons·m^–2^·s^−1^)	Temperature (°C)	pH
				O_2_ (mg/L)			
0.0	41.1674600	−112.6696117	149	5.46	2079	24.17	7.21
1.0	41.1674600	−112.6696117	151	5.38	1219	24.16	7.46
4.0	41.1674717	−112.6696117	143	5.13	175	23.77	7.83
6.0	41.1674717	−112.6696417	151	2.32	75	23.37	8.07
6.5	41.1674833	−112.6696200	203	1.68	44	20.20 ^b^	7.10 ^b^
8.0	41.1674817	−112.6696083	247	0.98	0	16.59	5.95

^a^: Previously reported in Meuser *et al*. [50]; ^b^: Data points were not measured, but were interpolated based on observed trends.

### 2.2. Abundance, Composition, and Diversity of hydA in GSL Water Column

hydA amplicons were not detected in surface samples (0.0 meters) or from samples collected from depths of 1.0 or 4.0 meters (Figure 2A). In contrast, hydA genes were detected in samples collected from depth intervals of 6.0, 6.5 and 8.0 meters, as well as from benthic sediment samples collected at 8.5 meters. The relative abundance of hydA templates, when normalized to the total quantity of extractable DNA, increased with increasing depth with the highest concentration of hydA genes (9.2 ± 1.9 × 10^5^ templates/ng DNA) detected in association with benthic sediment biomass (Table 2). The distribution of hydA along the DWR3 water column is coincident with the position of the thermocline and with decreasing levels of PAR and dissolved O_2_ and increasing salinity. The abundance of hydA templates was significantly and inversely correlated with temperature (Pearson R = −0.96, *p* < 0.01) and dissolved O_2_ (Pearson R = −0.85, *p* = 0.03) and was positively correlated with salinity (Pearson R = 0.93, *p* < 0.01).

**Figure 1:**
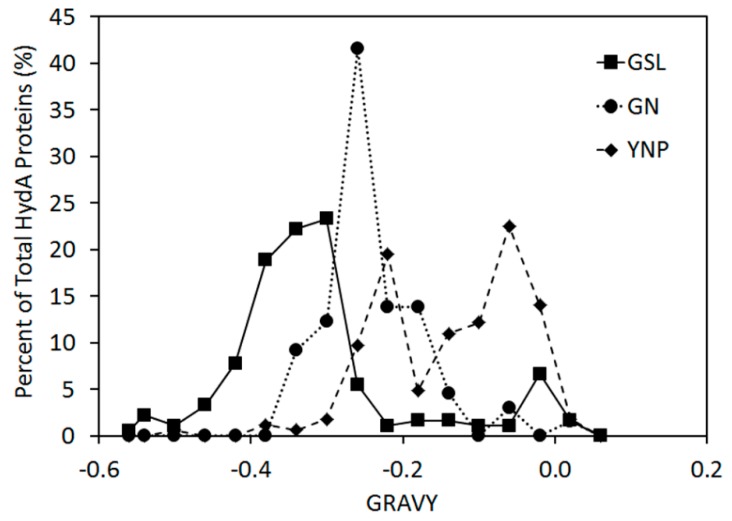
Hydropathy values associated with HydA from Great Salt Lake (GSL), Guerrerro Negro (GN), and Yellowstone National Park (YNP), as assessed using the GRAVY index (grand average hydropathy of amino acids). GRAVY indices for sequences from each environment were binned at 0.04 increments (rounded up).

A total of 211 *hydA* sequences (47 to 58 clones per sample) that, when translated, harbored signature motifs characteristic of HydA [3] were obtained in the current study (Table 2). The average hydropathy of amino acids (GRAVY index) comprising the inferred protein sequences was calculated and compared to sequences obtained with the same primer sets from other environmental systems, including microbial mat communities inhabiting salterns at Guerrero Negro (GN) [41] and hot springs in Yellowstone National Park (YNP), USA [40] (Figure 1). The GRAVY index for HydA sequences obtained from the GSL DWR3 water column spanned values ranging from 0.04 (indicative of average hydrophobic amino acid content) to −0.56 (indicative of very low hydrophobic amino acid content). There was no discernible pattern in the average GRAVY index for HydA sequences obtained from the GSL with sampling depth (data not shown). However, the average GRAVY index for sequences obtained from the GSL water column (14.9% to 24.7% salinity) was −0.30 whereas average GRAVY indices obtained from GN (8% salt) and YNP (0.5% to 3.1% salinity) were −0.26 and −0.15, respectively. This indicates that HydA from GSL, on average, comprise a greater number of hydrophilic amino acids than those from GN and YNP.

**Table 2:** Distribution, abundance, and phylogenetic diversity of *hydA* as a function of depth at the Great Salt Lake (GSL), Utah Division of Wildlife Resources sample site number 3 (DWR3) vertical transect.

Depth (m)	Templates/ng DNA	SD	*n* ^a^	PD ^b^
0.0	BD	−	−	−
1.0	BD	−	−	−
4.0	BD	−	−	−
6.0	1.6 × 10^5^	1.0 × 10^4^	58	12.5
6.5	1.9 × 10^5^	3.0 × 10^4^	55	12.5
8.0	5.9 × 10^5^	3.1 × 10^4^	53	13.9
Benthic ^c^	9.2 × 10^5^	1.9 × 10^4^	59	16.0

^a^: The total number of *hydA* sequences obtained from a given depth; ^b^: Faith’s index of HydA phylogenetic diversity; ^c^: Benthic sediment was obtained from a depth of 8.5 meters. Abbreviations: BD, below detection; SD, standard deviation.

**Figure 2:**
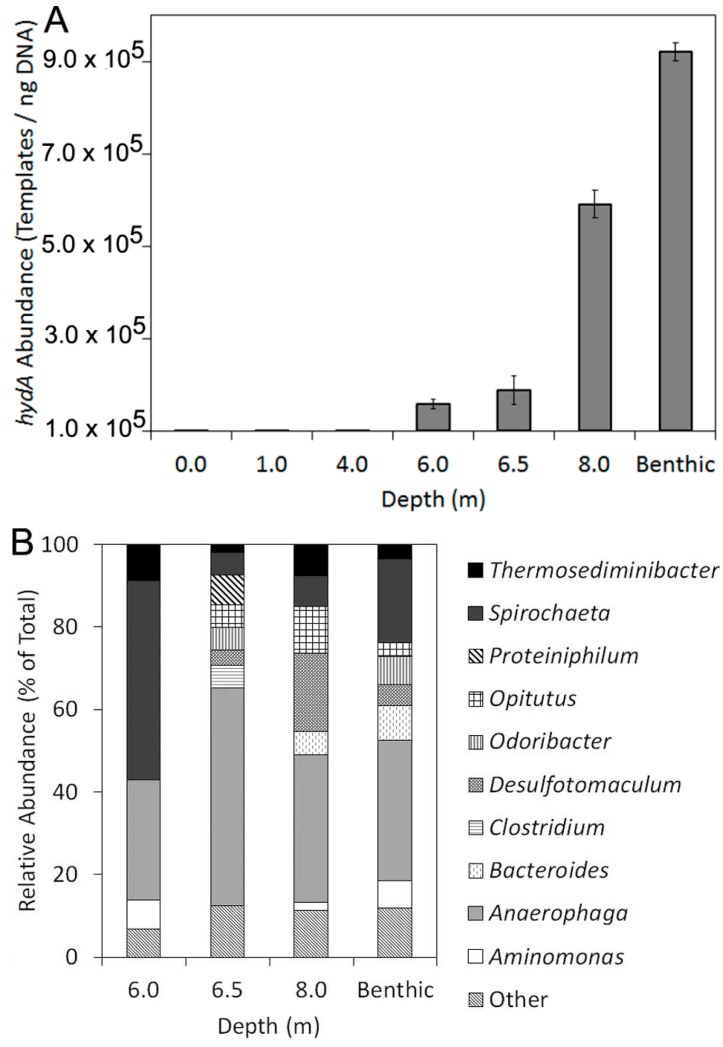
Abundance of *hydA* templates as a function of depth at DWR3 (**A**); Taxonomic composition of HydA protein sequences as determined by BLASTp analysis (**B**). Further details of the taxonomic composition of HydA are located in Supplementary Table S1.

Faith’s index of phylogenetic diversity (PD), a diversity metric that quantifies the proportion of total branch length in the phylogeny associated with sequences obtained from a given environment when compared to all environments when considered together, increased systematically for HydA assemblages with increasing depth (Table 2). HydA deduced amino acid sequences recovered from the four GSL environments were distantly related to HydA from cultivated organisms, with the average, minimum, and maximum observed sequence identities of 64%, 52%, and 76% (Supplementary Table S1). The majority (99.6%) of the sequences obtained from the 4 GSL environments exhibited affiliation with bacteria, although a single sequence affiliated with a eukaryotic diatom *Thalassiosira pseudonana* (62% sequence identity) was recovered from the benthic sediments. Despite significant variation in the taxonomic affiliation of HydA assemblages sampled at depth, binning the sequences at the class level did not reveal patterns in the taxonomic composition of assemblages as a function of depth (Figure 2B), consistent with previous studies indicating the tendency for *hydA* to be horizontally transferred which would likely obscure such patterns [42]. At the order level, HydA sequences affiliated with the *Clostridiales* were most abundant across all of the sample transects and at the genus level, HydA sequences most closely related to *Anaerophaga* were the most abundant regardless of sample depth.

### 2.3. Variation in H-Cluster Binding Motifs and Putative Gas Channels

**Figure 3:**
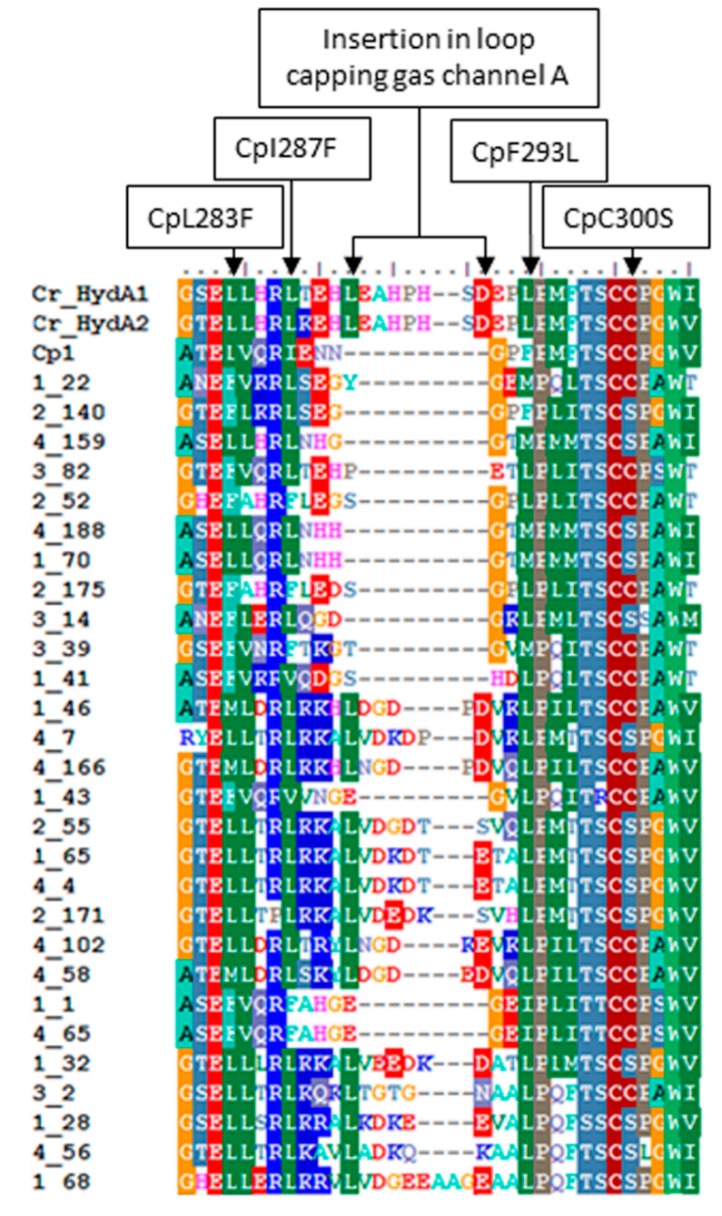
Unique substitutions in the L1 motif and insertions upstream from the L1 motif observed in recovered GSL HydA protein fragments. HydA1 and HydA2 from *C. reinhardtii* (Cr_HydA1 and Cr_HydA2, respectively) and HydA1 from *C. pasteurianum* (Cp1) are indicated. Substitutions and insertions are outlined above in reference to the Cp1 sequence.

Amino acid sequence alignments of the DWR3 HydA sequences with sequences of well-characterized HydA revealed a variety of substitutions in the L1 motif, a series of highly-conserved H-cluster binding residues. The observed substitutions included a Cys to Ser substitution relative to position 300 of the *C. pasteurianum* (Cp1) HydA sequence (AAA23248). This residue is involved in coordinating the active site H-cluster [10]. Interestingly, a number of substitutions were also observed in residues that line a putative gas channel that leads to the O_2_ sensitive active site H-cluster (Figure 3) [45]. In particular, substitution of the much larger Phe for Lys and Ile (positions 283 and 287 of Cp1, respectively) were noted in several GSL sequences. Conversely, other proteins exhibited substitution of Lys for Phe (position 293 of Cp1). Intriguingly, a large insertion just upstream from the L1 motif was observed in a number of sequences recovered from GSL [45]. Similar insertions are common in phototrophic algae, although it is unclear what functional significance (if any) that they may have [51].

### 2.4. Inferred Structural Variation of GSL HydA

**Figure 4:**
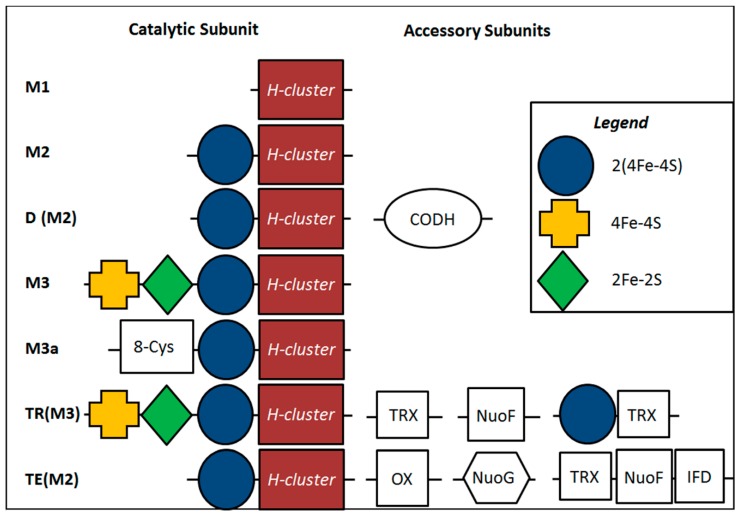
Accessary cluster domains associated with diverse HydA. The composition of *N*- and *C*-terminal modules (F- and C-clusters, respectively) as identified in Meyer *et al*., 2007 [3] and as modified by Calusinska *et al*., 2010 [19] are presented. Abbreviations: M, monomeric; D, dimeric; TR, trimeric; TE, tetrameric; CODH, carbon monoxide dehydrogenase module; TRX, thioredoxin domain; OX, oxidoreductase domain; NuoF, NuoF of complex I domain; NuoG, NuoG of complex I domain; and IFD, indolepyruvate: Fd domain.

**Figure 5:**
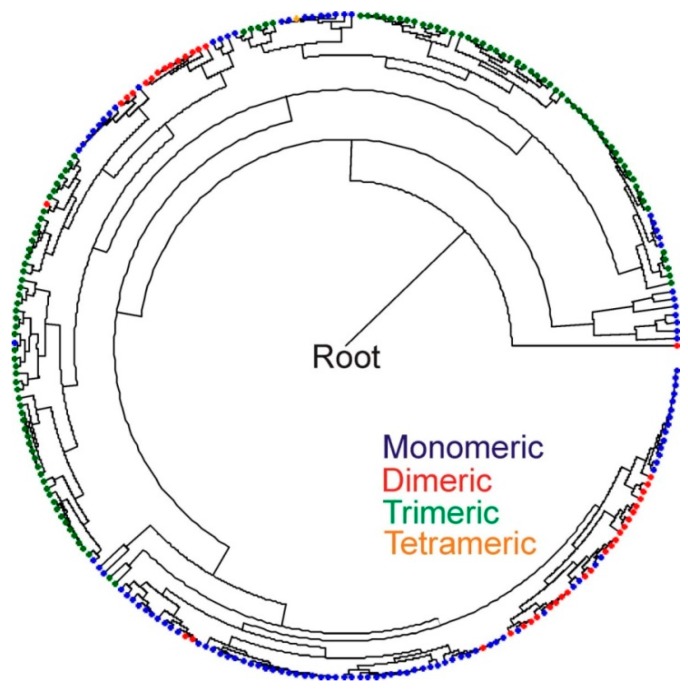
Bayesian phylogenetic reconstruction of 263 HydA protein sequences compiled from Calusinska *et al*., 2010 [19] and from GenBank (Supplementary Table S3). The phylogeny was generated using only the region of HydA corresponding to the fragment obtained from the GSL water column and sediments (positions 280 to 419 of HydA1 from *C. pasteurianum* (AAA23248)). The primary quaternary structure of the homologs (monomeric, dimeric, trimeric, tetrameric) as outlined in Meyer *et al*., 2007 [3] and more recently in Calusinska *et al*., 2010 [19] are overlain on the phylogeny. These primary quaternary structural architectures correspond with those presented in Figure 4.

A database of HydA sequences (Supplementary Table S3) was compiled from complete genome sequences present in GenBank based on previous characterizations of their *N*- and *C*-terminal cluster composition (Figure 4) and quaternary structure [3,19]. Additional sequences were obtained based on BLASTp searches and the primary sequence and quaternary structural characteristics were determined as described previously [19]. These sequences and the modular composition of the *N*- and *C*-terminal domains (*i.e.*, F- and C-clusters) and their quaternary structure was used to develop a Bayesian phylogenetic framework for predicting these features based on phylogenetic clustering of the HydA fragment amplified with the primers used herein. Similar to previous phylogenetic analyses of the H-cluster domain [3,19], phylogenetic reconstruction of just the H-cluster fragment (positions 280 to 419 of HydA1 from Cp1) in sequences recovered from GenBank revealed clustering that corresponded to primary and quaternary structural characteristics (Figure 5). This indicates that the phylogenetic signal of the amplified fragment is sufficient for predicting the characteristics of HydA from GSL at these levels of consideration. The majority (93.8% of total sequences) of HydA homologs recovered from GSL were nested within or formed clusters with reference sequences that have the M3 architecture and that are likely to form trimers (TR(M3)) (Supplementary Table S1). Only a small fraction of sequences were inferred to have a thermotogae monomeric configuration (5.3% of total sequences). No discernible pattern was observed in the distribution of these sequences as a function of depth in the DWR3 water column (data not shown). A small number of HydA sequences (0.88% of the total) did not cluster or nest phylogenetically with sequences comprising our database preventing prediction of their F- and C-cluster composition or quaternary structure. These sequences generally had low sequence identities to known HydA in genomic databases.

## 3. Discussion

A diversity of [FeFe]-hydrogenase were identified along the DWR3 vertical gradient in the GSL. The phylogenetic framework developed here indicates that the majority of HydA sequences from GSL form trimers and thus are possibly involved in H_2_-based electron bifurcation; however, it is unclear if they are involved in the production or consumption of H_2_. Examples of bifurcating [FeFe]-hydrogenase involved in oxidation and production of H_2_ have been described. For example, an enzyme that functions to bifurcate electrons derived from the oxidation of H_2_ coupled to the reduction of Fd and NAD^+^ has been identified in *A.* *woodii* [52] while other enzymes that function to couple the oxidation of Fd and NADH to the formation of H_2_ have been identified in *T.* *maritima*, *T.* *saccharolyticum*, and *T.* *tengcongensis* [21,23,53]. Moreover, two recent studies reveal the presence of reversible bifurcating complexes. In the case of the acetogen *M.* *thermoacetica*, a reversible Fd- and NAD-dependent trimeric [FeFe]-hydrogenase was identified [27].

Most organisms that harbor a bifurcating [FeFe]-hydrogenase harbor additional non-bifurcating enzymes [3,19]. It is therefore a surprise that the majority of sequences recovered in the present examination of HydA diversity in GSL are predicted to be of the bifurcating type. It is possible that the primers used in the present study are biased toward homologs that form trimers and that are thus potentially involved in bifurcation (e.g., TR(M3)) [3,19]. Indeed, a prior comparison of the primers used in this study with other primer designs found that each is biased toward a different spectrum of *hydA* diversity [42]. Application of untargeted shotgun metagenomic sequencing approaches, which are currently ongoing, will provide a mechanism to: (1) evaluate the potential bias associated with the recovery of predominantly bifurcating enzymes in the present study; and (2) evaluate the predictive capacity of the phylogenetic approaches used here to assign secondary and quaternary structure to the recovered protein sequences. Regardless, the recovery of a number of diverse HydA sequences from the GSL water column, including many exhibiting key amino acid substitutions, indicates a role for the putative bifurcating enzymes *in situ*.

The distribution of genes encoding putative bifurcating [FeFe]-hydrogenase was strongly influenced by gradients of both O_2_ and salinity. High salinity, such as that observed along the DWR3 vertical gradient, is generally unfavorable to proteins due to lower water activity, increases in the strength of hydrophobic interactions during protein folding, and a reduction in electrostatic interactions in proteins residing in the cytoplasm [54,55]. HydA recovered from the hypersaline GSL (15%–24% salt) water column and sediments were enriched in hydrophilic amino acids as indicated by significantly lower GRAVY indices when compared to HydA sequences obtained from lower salinity mats inhabiting GN salterns (8.0% salt) and YNP hot springs (0.5%–3.1% salt). This finding is consistent with the results of previous investigations of the proteomes of a variety of halophilic and non-halophilic organisms which indicate lower hydrophobicity indices than for non-halophilic counterparts. Lower hydrophobicity is a feature that is hypothesized to reduce the effects of salt-driven non-specific inter- or intra-molecular interactions in hydrophobic side-chains, which may lead to protein mis-folding and/or aggregation [54,56,57,58,59,60]. Similar results—lower hydrophobicity in proteins from halophilic organisms—were obtained from a comparative genomic and proteomic analysis of 6 halophilic and 24 non-halophilic isolates, spanning both the bacterial and archaeal domains [61]. Thus, the lower hydrophobicity observed in GSL HydA sequences suggests that these proteins are uniquely adapted to the high salt conditions present in their natural environment.

HydA was identified in transects of the DWR3 water column where salt levels were high (15%–24%) but where dissolved O_2_ was depleted relative to surface waters (dissolved O_2_ range 0.98–2.32 mg/L). The hypoxic nature of the DWR3 transects where *hydA* was detected implies either anaerobic microniches that allow anaerobes to exist in otherwise sub-oxic conditions, a lower effective exposure of the organism or its O_2_-sensitive enzymes to O_2_, or enhanced tolerance to O_2_. The solubility and bioavailability of O_2_ is lower in hypersaline solutions [62], which may allow anaerobic organisms to inhabit otherwise hypoxic conditions. Intriguingly, many of the HydA sequences recovered in this study exhibit novel substitutions in the L1 sequence motifs which are involved in the coordination of the O_2_-labile [4Fe-4S] subcluster of the H-cluster of HydA [10,63,64]. Substitutions in this region may have implications for the redox properties of this [4Fe-4S] cluster and thus may be important in conferring stability in the presence of O_2_ [11].

In particular, substitution of Ser for Cys in the L1 motif was commonly observed both in the GSL HydA fragments as well as HydA fragments obtained from GN [41]. Ser coordination of Fe-S clusters is uncommon in nature. The only well-characterized enzyme displaying such coordination is the P-cluster of nitrogenase where the Ser is thought to stabilize the cluster in its oxidized state [65,66]. It is possible that the L1 motif Cys-to-Ser variant observed in a number of the GSL and GN HydA sequences (corresponding to position 300 in the [FeFe]-hydrogenase from *C. pasteurianum*) may also have a stabilizing effect on the H-cluster, functioning to stabilize a more oxidized state of the active site in the presence of O_2_.

The variations in the residues forming the gas channel in the GSL HydA sequences may also play a role in conferring O_2_ tolerance. The most striking variations, CpL283F and CpI287F (positions in reference to CpHydA; Figure 3) may help to act as a molecular sieve [45,67]. Here, the larger sized side chain of Phe is expected to effectively decrease the rate of O_2_ diffusion but have little effect on rates of H_2_ diffusion due in principle to the smaller size of H_2_ relative to O_2_. Lastly the large insertion observed in numerous HydA homologs identified in GSL near the opening of gas channel A may also function as a molecular sieve or could even act as cap, both of which may slow diffusion of O_2_ into the enzyme.

The detection of abundant putative trimeric bifurcating [FeFe]-hydrogenase along the GSL DWR3 vertical gradient coupled with the numerous studies that continue to expand the functional capabilities of these enzymes [23,26,27,52] may have a significant application in biofuel production. One limitation for the production of highly reduced bioproducts is that a large proportion of the metabolic electron flow is directed towards reduction of pyridine nucleotides (*i.e.*, to NAD(P)H). Thermodynamically the reduced pyridine nucleotides are not sufficient in energy for the production of a number of desirable, highly reduced biofuel products. [FeFe]-hydrogenase catalyzed electron bifurcation potentially overcomes this thermodynamic challenge by combining electrons from the reduced pyridine nucleotides with other lower potential electrons from reduced Fd [23,25,26,27]. This increases the reducing potential of electrons from reduced pyridine nucleotides such that a larger flux is directed toward the production of desired biofuels (*i.e.*, reduced products). That other electron donors (e.g., CO, formate) [26] can potentially feed into these complexes expands the potential use of these enzymes in directing the flux of metabolic electrons toward desired biofuels. Efforts aimed at isolating representatives of the dominant, yet uncharacterized organisms harboring the unique [FeFe]-hydrogenase enzymes identified herein should continue to expand our understanding of the diversity of biochemical reactions catalyzed by this unique class of enzyme, which may prove useful in overcoming key bioengineering barriers.

## 4. Experimental Section

### 4.1. Site Description and Sample Collection

Samples were collected from the Division of Wildlife Resources site 3 (DWR3) (Latitude 41.16746, Longitude−112.6696117) in June 2007 (surface, 1.0, 4.0, 6.0, 6.5 and 8.0 meters depths and benthic sediments), by lowering a 2 L Kemmerer collection bottle (Wildlife Supply, Yulee, FL, USA) to each depth sampled, with deionized water rinses between each sampling, as described previously [50]. Respective samples were transferred to sterile bottles leaving a minimum of gaseous headspace and then immersed in ice until further workup in the laboratory. Cells were then collected by centrifugation (4000× *g* for 15 min at 3 °C) and the supernatant removed. Cell pellets were resuspended in 1 mL of supernatant and the resuspension was frozen at −80 °C until used for DNA extraction.

### 4.2. Physical and Chemical Analysis

Chemical and physical measurements were compiled as a part of a previous study conducted on the DWR3 water column [50]. Briefly, photosynthetic active radiation (PAR, 400–700 µmol·photons·m^−2^·s^−1^) was determined using a Li-Cor LI-193 underwater spherical quantum sensor (Li-Cor Biosciences, Lincoln, NE, USA) and Li-Cor LI-250A light meter (Li-Cor Biosciences). A Troll 9500 multi-sensor (In-Situ Inc., Fort Collins, CO, USA) was used to determine pH, temperature, salinity, and dissolved O_2_.

### 4.3. DNA Extraction, Amplification, Cloning, and Sequencing of hydA

Total DNA was extracted from 200 mg aliquots of biomass using the PowerSoil DNA Isolation Kit (MoBio Inc., Carlsbad, CA, USA) following the manufacturer’s instructions. DNA was quantified fluorometrically as previously described [67]. All DNA extracts were screened for the presence of 16S rRNA genes to ensure the presence of PCR-amplifiable DNA using 10 ng of DNA and primers bacterial 1070F (5'-ATGGCTGTCGTCAGCT-3') and universal 1492R (5'-GGTTACCTTGTTACGACTT-3') [68]. Approximately 500 bp fragments of *hydA* were PCR-amplified in triplicate from 10 ng of environmental genomic DNA as template with primer pair FeFe-272F and FeFe-427R using previously established reagent concentrations and reaction conditions [41]. Equal volumes of each replicate amplification were pooled and purified using the Wizard PCR Preps DNA purification system (Promega, Madison, WI, USA), quantified using the Low DNA Mass Ladder (Invitrogen, Carlsbad, CA, USA), cloned using the pGem-T Easy Vector System (Promega), and sequenced using the M13F-M13R primer pair as previously described [69]. A total of 215 *hydA* gene sequences were sequenced in the present study and representatives of each phylotype (Supplementary Table S1) have been deposited in the GenBank, DDBJ, and EMBL databases under the accession numbers HM636647–HM636798.

### 4.4. Primary Sequence Analysis

MEGA (version. 4.0.1) [69] was used to translate and align *hydA* sequences specifying the Gonnet 250 protein weight matrix with a pairwise alignment gap opening penalty of 13 and gap extension penalty of 0.05. The aligned HydA sequences were screened for the presence of the L1 ((FLI)**T**S**C**(C/S)**P**(GAS)W(VIQH)) and L2 ((IVLF)M**PC**x(ASRD)**K**(KQ)xE) (conserved residues are in bold and underlined, and bracketed positions indicate “semiconserved” residues at that position) as outlined previously [3,5]. ClustalX [70] was also used to create a pairwise sequence identity matrix which was subsequently imported into DOTUR [71] to identify and group operational taxonomic units (OTUs) and to perform rarefaction analyses at a sequence identity threshold of 0.01. Indices describing the hydrophobicity of inferred protein sequences (Aliphatic Index (AI) and Grand Average of Hydropathicity (GRAVY)) were computed using the ProtParam tool available on the ExPASy proteomics server (http://au.expasy.org/tools/protparam.html) [72]. The AI is the relative volume occupied by aliphatic amino acid side chains (alanine, valine, isoleucine, and leucine) in a given protein or protein fragment and is calculated according to the formula:

Aliphatic index = *X*_(Ala)_ + *a* × *X*_(Val)_ + *b* × (*X*_(Ile)_ + *X*_(Leu)_) (1)

where *X*_(Ala)_, *X*_(Val)_, *X*_(Ile)_, and *X*_(Leu)_ are mole percent (100 × mole fraction) of alanine, valine, isoleucine, and leucine and *a* and *b* are the relative volume coefficients for valine (*a* = 2.9) and Leu/Ile (*b* = 3.9) relative to alanine [73], with higher indices indicative of a higher content of aliphatic side chains in the protein. The GRAVY index is the sum of the hydropathy values for each amino acid in a sequence divided by the number of residues [73], with positive GRAVY indices indicative of hydrophobicity and negative GRAVY indices indicative of hydrophilicity. For comparison, aliphatic and GRAVY indices were computed for putative HydA previously recovered from microbial mats inhabiting salterns from GN (GenBank: FJ623894–FJ623958) [41] and from geothermal springs in YNP (GenBank: GU362773–GU362867) [40] (Supplementary Table S2). Intervals of 0.04 GRAVY index units were empirically selected for use in binning GRAVY indices for sequences recovered from the three geochemically-distinct environments (GSL, YNP, GN). Indices were rounded downward to the nearest 0.04 unit interval prior to binning. 

### 4.5. Prediction of Environmental HydA Accessary Cluster Composition

The sequences used to develop the F- and C-cluster classification and tertiary structure scheme developed by Meyer *et al*., 2007 [3] and further augmented by Calusinska *et al*., 2010 [19] were compiled and used to create a database for use in predicting the accessory cluster composition and tertiary structure of putative HydA fragments recovered from the GSL water column (Supplementary Table S3). Additional sequences representing the closest BLASTp hits for each OTU were also compiled and characterized for F- and C-cluster composition and tertiary structure using the criteria of Calusinska *et al*., 2010 [19] and the Conserved Domain Database as implemented with BLASTp [74]. The classification of these proteins, along with those reported by Calusinska *et al*., 2010 [19], are reported in Supplementary Table S3 and a schematic illustrating their F- and C-cluster structural variation is presented in Figure 3.

HydA protein sequences from GSL and from our database were aligned, trimmed to the length of the amplified protein fragment (positions 272 to 427 in HydA from *Clostridium pasteurianum*), and subjected to Bayesian phylogenetic reconstruction as described previously [40]. The C- and F-cluster composition and tertiary structure of environmental HydA was then predicted by phylogenetic clustering with HydA comprising our database. Sequences that formed lineages without a representative from our database were classified as “unknown” with respect to F- and C-cluster composition and tertiary structure. Alignments of HydA fragments of reference sequences used to construct the predictive framework can be obtained by email correspondence with the authors.

### 4.6. hydA Quantitative PCR (qPCR)

qPCR was used to estimate the number of *hydA* templates in GSL DNA extracts according to previously described methods [75]. Standard curves were generated from plasmid DNA containing *hydA* amplified from the GSL sample sites. Three plasmid clones were used in generating a standard curve that relates template copy number to threshold qPCR amplification signal. The threshold amplification signal as a function of copy number varied by less than 1 cycle for each of the three plasmid clones; thus, the standard curves generated using each plasmid clone were averaged for use in calculating the average template abundances and standard deviation in template abundances from replicate qPCRs. A standard curve was generated over 6 orders of magnitude ranging from 91 to 1.1 × 10^7^ copies of template per assay (*R*^2^ = 0.995).

qPCR assays were performed in a Rotor-Gene 300 quantitative real-time PCR machine (Qiagen, Valencia, CA, USA) in 0.5 mL optically clear PCR tubes (Qiagen) using a SsoFast EvaGreen Supermix qPCR Kit (Bio-Rad Laboratories, Hercules, CA, USA). Assay reactions were amended to a final concentration of 0.4 mg·mL^−1^ molecular-grade bovine serum albumin (Roche, Indianapolis, IN, USA). qPCR cycling conditions were as follows: Initial denaturation (95 °C for 10 min) followed by 40 cycles of denaturation (95 °C for 10 s), annealing (56.5 °C for 15 s), and extension (72 °C for 20 s). Specificity of the qPCR assays was verified by melt curve analysis. The reported template abundances are the average and standard deviation of qPCR assays performed in triplicate.

### 4.7. HydA Phylogenetic Diversity

The phylogenetic position of *hydA* homologs was assessed using PhyML-aBayes [76] with the nuclear prelamin A recognition factor-like protein-encoding gene (XM_414836), a distantly related homolog of *hydA* [3], from *Gallus gallus* serving as the outgroup. The General Time Reversible (GTR) substitution model with gamma-shaped rate variation with a proportion of invariable sites was specified in the calculation, as recommended by Modeltest (version 3) [77]. The consensus phylogram was rate-smoothed using the multidimensional version of Rambaut’s parameterization as implemented in PAUP (version 4.0) [78]. Rate-smoothing was performed according to the parameters identified using Modeltest (version 3). This included the identification of the substitution model, the gamma distribution of rate variation across sites, the proportion of invariant sites, nucleobase frequencies, and the rate matrix for each phylogram. Phylocom (version 4.0.1) [79] was used to calculate Faith’s index of phylogenetic diversity (PD) using the rate smoothed Bayesian chronogram. PD is the proportion of total branch length in the phylogeny associated with the taxa in a given sample or assemblage. A higher PD index for an assemblage is indicative of higher phylogenetic diversity (phylogenetic richness) relative to the total sequence pool.

## Supplementary Materials

Supplementary tables can be found at http://www.mdpi.com/1422-0067/15/12/21947/s1.
